# SNP discovery and association study for growth, fatness and meat quality traits in Iberian crossbred pigs

**DOI:** 10.1038/s41598-022-20817-0

**Published:** 2022-09-30

**Authors:** C. Óvilo, N. Trakooljul, Y. Núñez, F. Hadlich, E. Murani, M. Ayuso, C. García-Contreras, M. Vázquez-Gómez, A. I. Rey, F. Garcia, J. M. García-Casco, C. López-Bote, B. Isabel, A. González-Bulnes, K. Wimmers, M. Muñoz

**Affiliations:** 1 Departamento Mejora Genética Animal, INIA-CSIC, Madrid, Spain; 2 Research Institute for Farm Animal Biology, FBN, Dummerstorf, Germany; 3 CoPeD, Department of Veterinary Sciences, University of Antwerp, Wilrijk, Belgium; 4 Department of Nutrition and Sustainable Animal Production, CSIC, Granada, Spain; 5 Sorbonne Université, INSERM, NutiOmique, Paris, France; 6 Departamento de Producción Animal, Facultad de Veterinaria, UCM, Madrid, Spain; 7 Facultad de Medicina Veterinaria, Universidad Cardenal Herrera-CEU, Valencia, Spain

**Keywords:** Genetics, Animal breeding, Genetic association study, Genetic markers, Genome, Genomics, Quantitative trait, Sequencing

## Abstract

Iberian pigs and its crosses are produced to obtain high-quality meat products. The objective of this work was to evaluate a wide panel of DNA markers, selected by biological and functional criteria, for association with traits related to muscle growth, fatness, meat quality and metabolism. We used 18 crossbred Iberian pigs with divergent postnatal growth patterns for whole genome sequencing and SNP discovery, with over 13 million variants being detected. We selected 1023 missense SNPs located on annotated genes and showing different allele frequencies between pigs with makerdly different growth patterns. We complemented this panel with 192 candidate SNPs obtained from literature mining and from muscle RNAseq data. The selected markers were genotyped in 480 Iberian × Duroc pigs from a commercial population, in which phenotypes were obtained, and an association study was performed for the 1005 successfully genotyped SNPs showing segregation. The results confirmed the effects of several known SNPs in candidate genes (such as *LEPR*, *ACACA, FTO, LIPE* or *SCD* on fatness, growth and fatty acid composition) and also disclosed interesting effects of new SNPs in less known genes such as *LRIG3*, *DENND1B*, *SOWAHB, EPHX1* or *NFE2L2* affecting body weight, average daily gain and adiposity at different ages, or *KRT10*, *NLE1*, *KCNH2* or *AHNAK* affecting fatness and FA composition. The results provide a valuable basis for future implementation of marker-assisted selection strategies in swine and contribute to a better understanding of the genetic architecture of relevant traits.

## Introduction

Iberian pig is an autochtonous fatty breed characterized by a high adipogenic potential, high appetite and outstanding meat quality. These features are consequence of their genetics and the traditional extensive production system, both contributing to the deposition of subcutaneous and intramuscular fat with a high content of oleic acid and antioxidants^1,2^. Iberian × Duroc crossbred pigs are the main genotype employed to produce Iberian meat products in intensive production systems, although their sensorial meat quality is considered lower than that of pure Iberian pigs^3–5^. Furthermore, crossbred Iberian pigs are characterised by very heterogeneous developmental, productive and quality traits^6^, with great potential to identify the underlying genes. Knowledge of the molecular genetic basis of relevant traits would help in the design of marker-assisted selection programs.

Different candidate gene studies have been performed in Iberian pig populations, mostly in purebred animals or experimental crosses and with low-scale structural approaches, in order to deepen in the genetic architecture of productive traits. Relevant genes and mutations have been identified so far with this approach, mainly those detected in the IBMAP experimental populations such as *LEPR*^7,8^, *ELOVL6*^9^ or *ACSL4*^10,11^. Recently, Fernandez-Barroso et al.^12^ found significant associations of mutations in *PRKAG3*, *CAPN1* and several other genes on meat quality traits in pure Iberian animals. Also, functional genetic studies have been performed in Iberian pigs which have provided information on functional candidate genes and pathways potentially involved in phenotypic variation^5,13–15^. On the other hand, the use of commercially available high-density SNP arrays for genome-wide association studies (GWAS) provides a systematic and powerful approach for deepening into the genetic basis of complex traits. This approach has led to the identification of many interesting genomic regions and candidate genes in different populations and breeds, including the Iberian^16–22^. Nevertheless, the applicability of commercial SNP arrays is limited because they only identify a fraction of genetic variation. This is due to the fact that they have been designed from SNPs detected in a few cosmopolitan breeds resulting in limited informativity and power, specially for the analysis of local breeds^22,23^. In contrast, whole-genome sequencing (WGS) can potentially detect all genetic variants, including causal ones^24^ and may allow the design of customized, highly-informative SNP panels, useful to uncover the genetic basis of relevant traits in local breeds. Such advances make genetic improvement achievable for relevant productive and quality traits in non-cosmopolitan pig breeds.

The objective of this work was to discover and evaluate a wide panel of DNA markers for association with traits related to muscle growth, fat deposition and composition, metabolism and meat quality in Iberian crossbred pigs. For this purpose, a medium density custom genotyping protocol was used, designed with SNPs obtained and selected from a combination of whole genome sequencing data, transcriptome sequencing data and a candidate gene approach.

## Material and methods

### Ethics statement

The study was performed according to the Spanish Policy for Animal Protection RD53/2013, which meets the European Union Directive 2010/63/UE about the protection of animals used in research. The experiment was specifically assessed and approved (report CEEA 2012/036) by the INIA Committee of Ethics in Animal Research, which is the named Institutional Animal Care and Use Committee (IACUC) for the INIA. The study was carried out in compliance with the ARRIVE guidelines (https://arriveguidelines.org/arrive-guidelines).

### Animals and phenotypes

We used an Iberian × Duroc crossbred population. The animals were housed at a commercial farm, Ibéricos de Arauzo 2004 S.L. (Zorita de la Frontera, Salamanca, Spain). A total of 477 crossbred piglets (50% females and 50% males) born from 47 third and fourth-parity Iberian sows were involved in this study. The Iberian sows were inseminated with cooled semen from Duroc PIC boars (Genus plc, UK). The sows were individually identified with an electronic ear tag and housed in groups until day 101 of pregnancy, when they were moved to individual pens until the end of the suckling phase. Sows and offspring were fed standard diets for the Iberian breed, adjusted to fulfil individual daily requirements, based on recommendations from De Blas et al*.*^25^. The piglets were individually tagged at birth and remained with the sows until weaning. Within 2 days of birth, litters were equalled by cross-fostering and male piglets were surgically castrated. Surgical orchidectomy was performed after sedation with azaperone (1 mg/kg LW; Stresnil^®^, Ecuphar Veterinaria, S.L.U, Barcelona, Spain) and local anesthesia (lidocaine hydrochloride 2% solution; Lidocaina Normon^®^, 20 mg/mL, Laboratorios Normon, Madrid, Spain) applied with a 25G needle inserted directly into the spermatic cord through the skin. Piglets were weaned at an average age of 24 days-old, and housed in groups of 12 piglets/pen distributed by sex and body weight (BW) during the transition phase. In the growing-fattening phase (from 70 days-old until slaughter), animals were housed by sex and BW in groups of 40 pigs/pen. Females received immunocastration using VACSINCEL (Zoetis Inc., New Jersey, USA) with two vaccinations at 120 and 148 days-old, respectively. From day 240 until day 340 of age, pigs were marketed whenever they reached the minimum market weight, which for Iberian crossbred pigs is set at 115 kg of carcass weight. At day 340 of age, all remaining pigs were sent to market, independently of their weight. The phenotype evaluation followed the methods already described in a previous paper using part of the animals involved in this study^6^.

#### Evaluation of growth pattern and fatness

Different measurements were carried out at the following time points: birth, weaning (24 days-old), days 70, 110, 150, 180, 215 and 240 of averaged age and at slaughter. Body weight was determined individually at all these time points. Morphological measures (occipito-nasal length, biparietal diameter, trunk length, abdominal and thoracic perimeter and maximum thoracic diameter) were recorded at birth and at weaning for all piglets, with a measure tape. Backfat thickness and loin diameter were determined at weaning and at 110 and 215 days-old. Weight and length of carcasses and backfat thickness were recorded at the slaughterhouse.

The average daily weight gain (ADWG) was calculated during the suckling phase (1–24), the transition phase (25–70), and during the five selected time periods of the growing-fattening phase until the day 240 of age (70–110, 111–150, 151–180, 181–215 and 216–240 days of age; each period is named by its last day). The feed conversion ratio (FCR) was calculated by pen during the five selected periods of the growing-fattening phase. Average daily weight gain and FCR were also calculated for the whole periods from birth to slaughter and from weaning to slaughter, respectively.

#### Tissue sample collection and drip-loss analysis

Samples of blood were obtained in vivo (110 and 215 days-old) and at slaughter, and employed for DNA extraction and metabolic status evaluation. Samples of *longissimus dorsi* (LD) muscle, right liver lobe and subcutaneous fat were taken at slaughter and biobanked at − 20 °C until fatty acid (FA) composition was analysed. Muscle drip-loss analysis was also carried out^26^.

#### Evaluation of metabolic status

Metabolic status was assessed at the time points of 110 and 215 days-old and at slaughter. Blood samples were assayed for determination of parameters related to metabolism of glucose (glucose and fructosamine) and lipid profiles [total cholesterol, high-density lipoprotein cholesterol (HDL-c), low-density lipoprotein cholesterol (LDL-c) and triglycerides], by means of a clinical chemistry analyzer (Saturno 300 plus, Crony Instruments s.r.l., Rome, Italy).

#### Evaluation of fat content and FA composition of tissue samples

Liver fat (LF) and intramuscular fat (IMF) were extracted as described by Segura and Lopez-Bote^27^ and expressed as a percentage of dry matter. These lipids were fractionated into the two main fractions of the fat tissue: neutral lipids (NL) and polar lipids (PL)^1,27^. Subcutaneous fat was extracted and separated in outer and inner layers to be analysed individually. From individual FA values, proportions of saturated, monounsaturated, and polyunsaturated FA (SFA, MUFA, and PUFA), the unsaturation index (UI), and the sum of total n-3 FA (n-3) and n-6 FA (n-6) and its ratio (n-6/n-3) were calculated^28^. Moreover, the activity of stearoyl-CoA desaturase enzyme 1 (SCD1) was estimated using two desaturation indexes, the ratios of C18:1/C18:0 and MUFA/SFA^29^.

### SNP discovery

**Figure 1:**
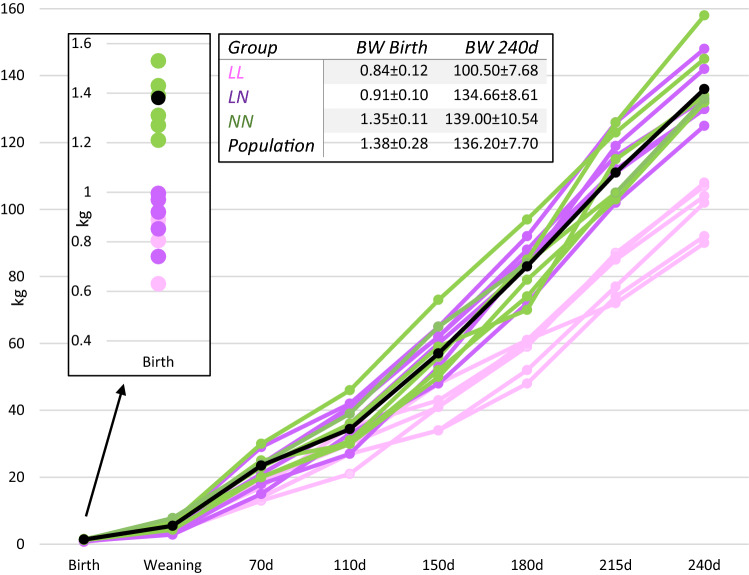
Growth patterns of the 18 animals selected for WGS. Six animals are included in each growth group: low birth weight and low end weight (LL; pink); low birth weight and normal end weight (LN; purple) and normal values for both birth and end weights (NN; green). Population mean is shown in black. *BW* body weight.

Out of the crossbred Iberian pig population, 18 male pigs with divergent postnatal growth patterns were selected for whole genome sequencing. Birth weight and BW at 240d were considered in order to select animals corresponding to three different growth categories: low birth weight and low final weight (LL; 6 animals), low birth weight but normal final weight (LN; 6 animals) and normal values for both birth and final weights (NN; 6 animals) (Fig. 1). Selected animals came from different litters.

Genomic DNA was obtained from the blood samples of all animals using the NucleoSpin^®^ Blood Kit (Macherey-Nagel, Düren, Germany). A NanoDrop 2000 spectrophotometer (Thermo Fisher Scientific, Waltham, Massachusetts, USA) was used to measure the concentration and quality of the DNA. Whole-genome resequencing of the selected 18 animals was performed on an Illumina HiSeq 2000 platform by CNAG-CRG (Barcelona, Spain). About 1 μg genomic DNA was randomly fragmented. The genomic library was prepared according to the manufacturer’s protocol (Illumina, True Seq DNA preparation guide) using the TruSeq DNA sample preparation kit. The paired-end library was sequenced on an Illumina HiSeq 2000 using the v4 chemistry and 2 × 125 reads.

FastQC software was employed to perform the quality control of the raw data (http://www.bioinformatics.babraham.ac.uk/projects/fastqc/). Trimmomatic package version 0.38^30^ was used for paired-end read trimming. Reads were trimmed to remove adapters, eliminate low quality or N-bases at the start or end of the read and scan the reads from the 3′-end with a 4 bases sliding window, cutting when the average Phred quality drops below 20. After trimming, reads were removed if they were shorter than 40 bp. Filtered paired reads were aligned to the porcine reference genome build 11^31^ by the Burrows-Wheeler Aligner (BWA version 0.7.10)^32^, employing the “bwa-mem” algorithm. SAMtools version 1.6^33^ was used to filter unmapped or unproperly mapped reads, sorting, indexing and adding read groups. Picard tools version 2.18.9 (http://broadinstitute.github.io/picard/) was employed to mark duplicates. From this step on till the Variant Call Format (VCF) file, the reads were processed using the Genome Analysis Toolkit (GATK version 4.0.6.0)^34,35^. Base quality was recalibrated using BaseRecalibrator and variant calling was carried out for each sample using HaplotypeCaller, resulting in Genomic Variant Call Format files (GVCFs), which were combined to a VCF file using CombineGVCFs using default parameter values. Finally, variants were called using GenotypeGVCFs using default parameters. A total of 14,871,405 and 3,613,138 raw SNPs and INDELS were detected, respectively. The SNPs were quality filtered using the GATK VariantFiltration tool, excluding those with parameters “QUAL < 40 || QD < 5.0 || FS > 60.0 || MQ < 40.0 || DP < 30” and resulting in 13,324,328 filtered SNPs. VCFtools^36^ was employed to filter those SNPs with Minor allele frequency (MAF) lower than 0.05, leading to a final set of 11,756,179 filtered SNPs.

Ensembl variant effect predictor (VEP ver. 99)^37^ was used to annotate the variants from our WGS dataset with information in the VEP database (the merged cache file Sscrofa11.1 ver. 99). The default parameter of --distance 5 kb of VEP was applied to define the upstream and downstream variants. We also applied --sift b option to the prediction of the SIFT score^38^ of missense variants.

### Selection of SNPs for genotyping

We focused on missense SNPs located on annotated genes and showing differences in allele frequencies between groups differing in growth patterns. A total of 32,688 markers were classified as missense out of the 11.7 million, according to VEP. LD-pruning was implemented with PLINK 1.9^39^ removing a total of 20,049 SNPs with r^2^ > 0.6 in 1000 kb windows. Finally, a total of 8419 SNPs mapped on autosomes were used for downstream analyses. Allele frequencies within growth groups were calculated with PLINK 1.9. We kept those SNPs having an allelic frequency difference between groups higher than 0.25 in at least one comparision (LL vs LN, LL vs NN or LN vs NN). As a result, a total of 2259 SNPs remained.

Among these preselected SNPs, 1023 were technically adequate for Agriseq genotyping. These were included in the design and genotyped by *Genotyping by Sequencing* (GBS) in the Iberian × Duroc population, from which phenotypes were also available. The list of markers genotyped by GBS is included as Supplementary Table S1. For GBS, libraries were made using Agriseq HTS library kit, and sequenced in Ion 540 chips on an Ion GeneStudio S5 Sequencer.

Besides, we complemented this panel with 192 candidate SNPs obtained from the literature mining and from pure and Duroc-crossbred Iberian muscle RNAseq data^13,14,40^ (Suplementary Table S2). These SNPs were genotyped in the same Iberian × Duroc population using a customed TaqMan^®^ OpenArray™ (OA) Genotyping platform (Thermo Fisher Scientific, Waltham, Massachusetts, USA). Genotyping was carried out in a QuantStudio™ 12 K Flex Real-Time PCR System (Thermo Fisher Scientific, Waltham, Massachusetts, USA) at the Veterinary Molecular Genetics Service (SVGM, UAB, Barcelona, Spain). DNA samples were loaded and amplified on the arrays following manufacturer instructions. Detection of allele-specific signal intensities was performed using OpenArray NT Imager, and the genotypes were called using OpenArray SNP Genotyping analysis software. In addition, SNPs images were visually inspected to detect any clustering issues.

### Statistical association analyses

The total SNP dataset (1215 markers) was filtered for association analyses using PLINK 1.9 by MAF value (0.1) and excluding markers with genotyping errors, with 1005 SNPs remaining after the filtering. These analyses were carried out on 477 individuals with genotyping and phenotyping data. SNP effects were analysed with the *leave-one-chromosome-out* option from the software GCTA^41^. This option implements a mixed linear model-based association analysis using the following general model:

$${\varvec{y}} = {\varvec{a}} + {\varvec{bX}} + {\varvec{fF}} + {\varvec{g}}{\text{-}} + {\varvec{e}}$$

where ***y*** is the phenotypic value corresponding to each trait, ***a*** is the mean term, ***b*** includes the additive effects (fixed term) of the analysed SNP, ***X*** includes the genotype indicator variable that is coded as 0, 1 or 2, ***f*** includes the remaining fixed effects and covariates that were different depending on the group of traits analysed (Suplementary Table S3) and ***F*** its corresponding incidence matrix, ***g-*** is the accumulated effect of all the SNPs (captured by the Genomic Relationship Matrix, GRM) except those mapped on the same chromosome than the SNP analysed and ***e*** is the residual. The genetic variance ***var*(*g-*)** is estimated based in the null model ***y* = *a* + *g-* + *e*** and then fixed while the association between each SNP and the trait is tested. This variance is re-estimated each time that a chromosome is excluded when the GRM is calculated. False discovery rate (FDR) was controlled for using the library q-value^42^ in Rstudio^43^. SNPs with a *p-*value and *q-*value lower than 0.05 and 0.10, respectively, were considered as significantly associated with the trait tested.

## Results

### SNP discovery and genotyping

An average of 83 million paired-reads were generated per sample by Illumina sequencing, ranging from 67 to 100 million, with a mean coverage of 8.4 ×. After quality control, 98% of the reads were mapped, on average, to the reference genome (Sscrofa11.1), and over 14 million variants were detected. After the application of several quality filters and functional and technical selection criteria, 1023 missense polymorphisms were finally genotyped by GBS.

GBS quality metrics confirmed the reliability of the genotyping procedure. Both average sample and marker call were 93.9%, and 90.2% of the markers had a call rate above 90%. Average coverage was 183 ×, well above the Agriseq recommended coverage of 100 × for genotyping purposes. Uniformity (percentage of target bases that have at least 0.2 times the mean depth) averaged 92.9%.

Out of the 1023 SNPs included in the GBS marker panel, 120 polymorphisms were discarded due to lack of segregation (MAF < 0.10) or genotyping errors, resulting in 903 GBS markers with useful data for the association study.

In parallel, 192 markers in candidate genes were genotyped by openarray. Out of them, 60 were monomorphic and a total of 30 discarded due to low variability (0 > MAF < 0.10) or genotyping errors, thus 102 OA markers remained informative.

**Figure 2:**
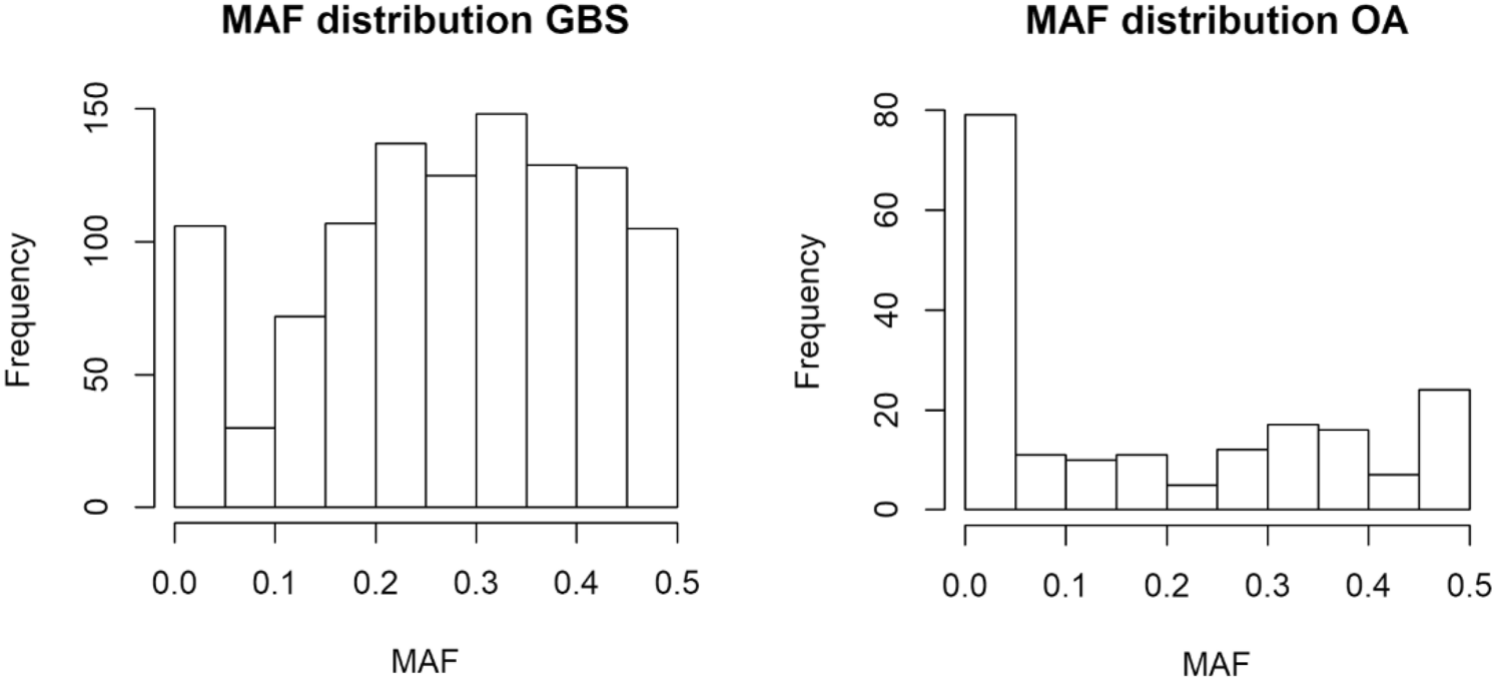
MAF distribution for markers genotyped by Genotyping by Sequencing (obtained from WGS data) or OpenArray (obtained from bibliography and RNAseq data).

Joint results of GBS and OA genotyping resulted in 1005 markers being included in the association study (903 and 102 from GBS and OA, respectively). Minimum allele frequencies (MAF) distribution for the genotyped markers in the Iberian crossbred population is shown in Fig. 2, which reflects a good proportion of markers with intermediate frequencies in the GBS marker panel coming from WGS data, and a much lower informativity in the OA panel coming from candidate genes proposed by either bibliography or RNAseq data.

### Association study

**Figure 3:**
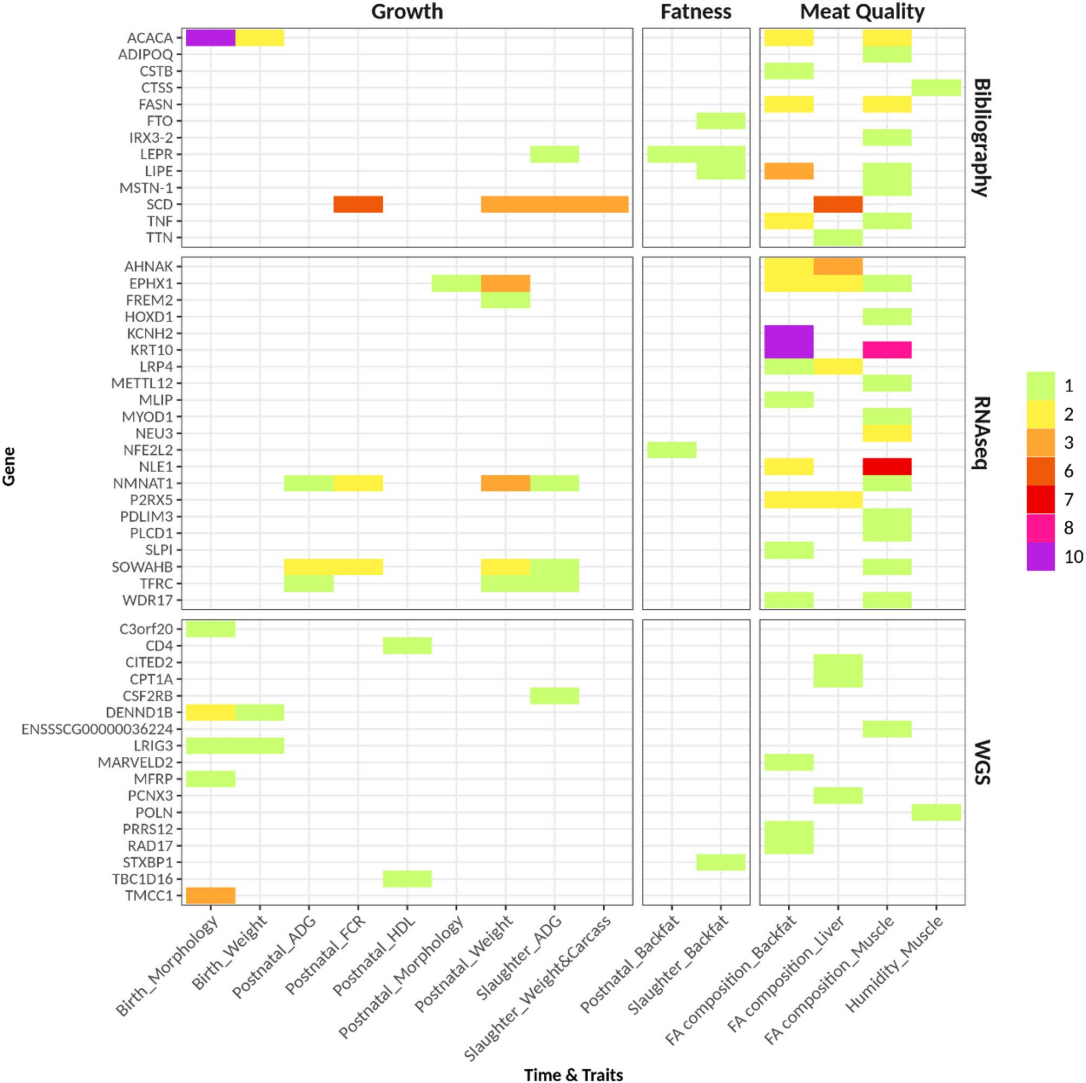
Summary of significant association results (q < 0.10) obtained for the different traits, grouped by trait type/stage (x axis) and source of polymorphisms (y axis). Colored bars denote the number of traits significantly associated with each gene. *ADG* average daily gain, *FCR* feed conversion ratio, *WGS* whole genome sequencing.

The association study was performed for the 1005 successfully genotyped SNPs showing segregation within our population and all growth, fatness, FA composition and metabolic traits available (descriptive statistics included in Supplementary Table S4). Significant association results (q < 0.10) are included in Supplementary Table S5 and a graphical representation of those results is shown in Fig. 3. Supplementary Table S6 includes association results with significant nominal *p*-values, for those genes showing at least one significant association (q < 0.10).

#### Growth and fatness

Regarding growth and fatness traits, 72 significant associations were observed, most of them involving candidate genes genotyped in the OA platform (n = 59) for which previous biological or functional hypothesis were available.

Candidate genes with SNPs affecting weight and morphological measures at early developmental stages (birth and weaning) included *ACACA*, *EPHX1* and *FREM2* genes (Supplementary Table S5). The two SNPs analysed within *ACACA* gene systematically affected all the body measures recorded at birth (Table 1). At weaning, punctual associations were detected for *EPHX1* and abdominal circumference, and also for *FREM2* and weaning weight, which reached the significance threshold. Several nominal-level associations were observed for other related traits in both genes (Supplementary Table S6). *FREM2* showed several suggestive associations with early development traits, while *EPHX1* showed suggestive associations with livelong growth and development traits. From 150 days-old, the main genes affecting weight, ADG and FCR were *NMNAT1*, *SOWAHB*, *EPHX1*, *TFRC* and *SCD*. Out of them, the three SNPs analysed within the *SCD* gene showed the effects with the largest magnitude, reaching, for instance, 29 kg (3.2 SD) for slaughter weight, 10 kg for carcass weight (1.25 SD) and 0.125 kg/d (1.9 SD) for global ADG (Table 2). A total of 13 associations involved SNPs detected in WGS and selected based on their potential effect on the coded protein and their difference in allele frequency between the growth groups. Results corresponding to the SNPs coming from WGS data provide new genes related to weight and body measures, only at birth, including *LRIG3*, *DENND1B*, *TMCC1* and *MFRP*. Among the genes associated with birth morphological traits, *ACACA* (Table 1) and *TMCC1* were those with the largest effects.

**Table 1:** Association results for polymorphism located in *ACACA* gene (75_*ACACA*, position 38,825,225). Traits with *p-*value < 0.01 are shown and those with q < 0.10 are highlighted in bold.

Time point/localization	Trait	b	SE	*p-*value	*q*-value
Birth	**Weight**	**− 0.137**	**0.029**	**3.33E−06**	**0.002**
	**Occipito-nasal lenght**	**− 0.310**	**0.071**	**1.18E−05**	**0.006**
	Trunk lenght	− 0.590	0.195	0.002	0.413
	**Biparietal diameter**	**− 0.132**	**0.032**	**4.11E−05**	**0.015**
	**Thoracic diameter**	**− 0.280**	**0.068**	**3.97E−05**	**0.024**
	**Abdominal circumference**	**− 0.758**	**0.173**	**1.23E−05**	**0.005**
	**Thoracic circumference**	**− 0.825**	**0.189**	**1.22E−05**	**0.007**
Weaning	Occipito-nasal lenght	− 0.227	0.088	0.010	0.829
Backfat, inner layer	C17:0	− 0.011	0.004	0.010	0.766
	C18:1(n-7)	− 0.208	0.069	0.003	0.866
Backfat, outer layer	C14:0	− 0.031	0.012	0.009	0.523
	**C16:1(n-7)**	**− 0.169**	**0.039**	**1.74E−05**	**0.007**
	C18:0	0.283	0.102	0.005	0.516
	MUFA	− 0.626	0.181	0.001	0.195
	MUFA/SFA	− 0.039	0.012	0.001	0.406
*Longissimus dorsi*, neutral lipids	C16:0	0.745	0.240	0.002	0.280
	**C16:1(n-7)**	− **0.333**	**0.077**	**1.44E−05**	**0.009**
	C17:0	− 0.009	0.004	0.010	0.626
	C17:1	− 0.019	0.006	0.001	0.345
	C18:0	0.408	0.122	0.001	0.350
	C18:1(n-9)	− 0.497	0.236	0.001	0.941
	C18:1(n-7)	− 0.211	0.065	0.001	0.345
	SFA	1.137	0.354	0.001	0.290
	MUFA	− 1.052	0.332	0.002	0.365
	UI	− 1.269	0.394	0.001	0.300
	C18:1/C18:0	− 0.281	0.087	0.001	0.348
	MFA/SFA	− 0.086	0.027	0.001	0.291
*Longissimus dorsi*, polar lipids	C22:4(n-6)	− 0.070	0.023	0.002	0.281
Liver, neutral lipids	C16:0	0.643	0.243	0.008	0.895

**Table 2:** Association results for polymorphisms in *SCD* gene (10_SCD, position 111,461,631). Traits with *p-*value < 0.01 are shown and those with q < 0.10 are highlighted in bold.

Timepoint, location	Trait	b	SE	*p-*value	*q-*value
150 days-old	Average daily gain	0.220	0.084	0.009	0.602
	**Feed conversion ratio**	**− 1.874**	**0.396**	**2.25E−06**	**0.001**
180 days-old	Average daily gain	0.205	0.067	0.002	0.238
	**Feed conversion ratio**	**− 2.541**	**0.469**	**5.90E−08**	**1.98E−05**
	Weight	20.587	6.545	0.002	0.208
215 days-old	Average daily gain	0.244	0.100	0.010	0.565
	Feed conversion ratio	− 1.990	0.709	0.005	0.669
	**Weight**	**28.859**	**8.348**	**0.001**	**0.078**
Slaughter	**Global average daily gain**	**0.125**	**0.035**	**4.04E−04**	**0.072**
	**Weight**	**29.318**	**5.454**	**7.63E−08**	**2.55E−05**
	Average daily gain	0.126	0.044	0.004	0.245
	Liver fat content	− 5.296	1.539	0.001	0.150
*Longissimus dorsi*, neutral lipids	C20:5(n-3)	− 0.135	0.047	0.005	0.503
Liver, neutral lipids	C14:0	− 0.548	0.154	3.84E−04	0.129
	C16:0	− 3.877	1.247	0.002	0.635
	**C16:1(n-9)**	**− 0.454**	**0.119**	**1.41E−04**	**0.048**
	C16:1(n-7)	− 0.920	0.280	0.001	0.349
	C17:1	− 0.114	0.036	0.001	0.504
	C18:0	7.225	2.257	0.001	0.458
	C18:1(n-9)	− 9.064	2.679	0.001	0.243
	C18:2 (n-6)	1.863	0.638	0.004	0.507
	C20:4(n-6)	4.363	1.457	0.003	0.706
	C20:5(n-3)	0.150	0.046	0.001	0.378
	C22:4(n-6)	0.252	0.098	0.010	0.990
	C22:6(n-3)	0.457	0.166	0.006	0.981
	MUFA	− 10.510	3.112	0.001	0.249
	PUFA	7.393	2.356	0.002	0.580
	UI	0.171	0.062	0.006	0.804
	n3	0.985	0.335	0.003	0.993
	n6	6.407	2.052	0.002	0.612
	**C18:1/C18:0**	**− 1.094**	**0.279**	**8.73E−05**	**0.030**
	MUFA/SFA	− 0.298	0.089	0.001	0.291

Backfat was affected by *LEPR*, *NFE2L2*, *FTO* and *LIPE* candidate genes (Supplementary Table S5). *LEPR* showed the most evident impact on backfat, with significant or suggestive effects on the depth of the different fat layers during the growing stage and several other traits (Table 3) and with the effect of largest magnitude at slaughter (0.7 SD). In addition, a SNP located in *CFAP157/ STXBP1*, derived from WGS data, was associated with backfat thickness at slaughter, with an effect of relevant magnitude (0.6 SD).

**Table 3:** Association results for *LEPRc*.1987C/T polymorphism (001_LEPR, position 146,829,589). Traits with *p-*value < 0.01 are shown and those with q < 0.10 are highlighted in bold.

Timepoint, location	Trait	b	SE	*p-*value	*q-*value
Weaning	Head lenght	− 0.428	0.157	0.007	0.735
110 d	Weight	− 2.828	1.030	0.006	0.349
	Backfat inner layer	− 0.067	0.023	0.003	0.427
	**Backfat**	**− 0.148**	**0.036**	**3.71E−05**	**0.037**
	HDL	− 17.424	6.256	0.005	0.891
150 d	Weight	− 4.451	1.606	0.006	0.294
215 d	Weight	− 7.794	2.658	0.003	0.260
	ADG	− 0.098	0.031	0.002	0.424
Slaughter	**Global ADG**	**− 0.040**	**0.011**	**4.29E−04**	**0.072**
	**Backfat**	**− 0.467**	**0.129**	**2.86E−04**	**0.072**
	Cholesterol	− 9.716	3.474	0.005	0.826
	HDL	− 3.518	1.349	0.009	0.993
	LDL	− 7.435	2.885	0.010	0.994
Liver neutral fraction	C20:0	− 0.099	0.038	0.009	0.772

#### Fatty acid composition

Fatty acid composition was determined in backfat (inner and outer layers) and in *Longissimus dorsi* muscle and liver tissues (neutral and polar lipid fractions). A total of 100 significant associations were found for these traits (Supplementary Table S5), out of which 45 associations corresponded to backfat FA composition (13 for the inner layer and 32 for the outer layer), 36 corresponded to loin FA (25 for neutral and 11 for polar lipid fractions) and 19 corresponded to liver FA profile (12 for neutral and 7 for polar lipid fractions). As for growth and fatness traits, most associations corresponded to markers located in biological and functional candidate genes genotyped by OA (n = 93) in comparison to those derived from WGS (n = 7). The 100 significant associations were related to 42 SNPs, located in 35 genes, and showing between 1 and 18 significant effects on different traits or tissues. Results for those SNPs having at least 2 significant effects are shown in Table 4. Genes with the highest number of significant associations with FA traits were *KRT10* (Tables 4 and 5), with 18 significant associations; and *NLE1*, with 9 significant associations (Tables 4 and 6), both genes affecting the FA profile of backfat and muscle neutral lipids, mainly regarding short chain saturated and monounsaturated FA. Variation in *KCNH2* gene was significantly associated with several main PUFA measures (C18:2n-6, C18:3n-3, PUFA, n-3, n-6) only in the backfat outer layer; while other genes such as *AHNAK*, *LIPE*, *EPHX1* or *TNF* had effects on different locations affecting similar traits (Table 4).

**Table 4:** Significant association results for FA composition traits, including SNPs with at least two significant effects on different traits or different tissues (q < 0.10).

Gene	Chr	Position	Backfat Inner	Backfat Outer	Loin Neutral	Loin Polar	Liver Neutral	Liver Polar	n associations
*KRT10*	12	21,641,370	C14:0, C16:0, SFA, MUFA, UI, MUFA/SFA	C14:0, C16:0, MUFA, UI	C14:0, C16:0, C18:1n-9, SFA, MUFA, UI, C18:1/C18:0, MUFA/SFA				18
*NLE1*	12	40,037,354	C18:0, MUFA/SFA		C16:0, C18:1n-9, SFA, MUFA, UI, C18:1/C18:0, MUFA/SFA				9
*EPHX1*	10	13,768,090	C20:1n-9	C20:1n-9	C20:1n-9		C20:3n-6	C20:3n-6	5
*KCNH2*	18	6,276,970 6,275,028		C18:2n-6, C18:3n-3, PUFA, n-3, n-6					5
*AHNAK*	2	9,198,996	C18:0	C16:1n-7			C20:3n-6	C18:2n-6, C20:3n-6	5
*LIPE*	6	49,558,911	C14:0	C14:0, C16:0	C14:0				4
*TNF*	7	23,701,215	C14:0	C14:0	C14:0				3
*LRP4*	2	15,651,335		C14:0				C18:2n-6, C20:3n-6	3
*SCD*	14	111,461,631 111,461,751 111,461,804					C16:1n-9, C18:1/C18:0		2
*FASN*	12	926,299 929,967		C14:0	C14:0				2
*ACACA*	12	38,825,225 38,824,185		C16:1n-7	C16:1n-7				2
*WDR17*	15	38,694,151	C20:1n-9			C20:3n-6			2
*P2RX5*	12	49,788,563 49,786,710		C14:0			C20:3n-6		2

**Table 5:** Association results for polymorphism in *KRT10* gene (175_KRT10, position 21,641,370). Traits with *p-*value < 0.01 are shown and those with q < 0.10 are highlighted in bold.

Timepoint/location	Trait	b	SE	*p-*value	*q-*value
Slaughter	Intramuscular fat	0.015	0.006	0.009	0.384
Backfat, inner layer	**C14:0**	**− 0.055**	**0.012**	**1.21E−05**	**0.012**
	**C16:0**	**− 0.583**	**0.117**	**5.55E−07**	**0.001**
	C18:1(n-9)	0.500	0.155	0.001	0.404
	C18:1(n-7)	0.172	0.060	0.004	0.912
	**SFA**	**− 0.912**	**0.215**	**2.17E−05**	**0.022**
	**MUFA**	**0.731**	**0.187**	**9.30E−05**	**0.093**
	**UI**	**0.010**	**0.003**	**9.69E−05**	**0.097**
	**MUFA/SFA**	**0.056**	**0.013**	**1.43E−05**	**0.014**
Backfat, outer layer	**C14:0**	**− 0.040**	**0.010**	**1.06E−04**	**0.016**
	**C16:0**	**− 0.560**	**0.101**	**2.89E−08**	**2.90E−05**
	C18:0	**− **0.247	0.091	0.007	0.516
	C18:1(n-9)	0.455	0.138	0.001	0.558
	C18:2(n-6)	0.170	0.062	0.006	0.859
	SFA	**− **0.644	0.174	2.17E-04	0.218
	**MUFA**	**0.675**	**0.162**	**3.10E−05**	**0.031**
	PUFA	0.183	0.067	0.007	0.844
	**UI**	**0.011**	**0.002**	**1.21E−06**	**0.001**
	n6	0.172	0.063	0.006	0.852
	MUFA/SFA	0.043	0.011	1.29E−04	0.129
*Longissimus dorsi* muscle, neutral lipids	**C14:0**	**− 0.064**	**0.013**	**6.87E−07**	**0.001**
	**C16:0**	**− 1.203**	**0.209**	**8.73E−09**	**8.76E−06**
	C16:1(n-7)	0.197	0.068	0.004	0.221
	C17:1	0.013	0.005	0.009	0.481
	C18:0	**− **0.276	0.107	0.010	0.471
	**C18:1(n-9)**	**1.022**	**0.205**	**6.18E−07**	**0.001**
	C18:1(n-7)	0.172	0.057	0.002	0.350
	C18:2(n-6)	0.131	0.051	0.010	0.770
	**SFA**	**− 1.541**	**0.307**	**5.28E−07**	**0.001**
	**MUFA**	**1.394**	**0.288**	**1.33E−06**	**0.001**
	**UI**	**1.702**	**0.342**	**6.67E−07**	**0.001**
	n6	0.137	0.053	0.010	0.776
	**C18:1/C18:0**	**0.288**	**0.076**	**1.44E−04**	**0.072**
	**MUFA/SFA**	**0.120**	**0.024**	**3.45E−07**	**3.46E−04**
*Longissimus dorsi*, polar lipids	C16:0	− 0.373	0.107	0.001	0.198
	C20:3(n-6)	0.039	0.015	0.009	0.401
	C22:5(n-3)	0.058	0.021	0.007	0.921
	SFA	− 0.412	0.145	0.005	0.881
	n3	0.082	0.031	0.008	0.582
	n6/n3	− 0.299	0.106	0.005	0.646
	MFA/SFA	0.018	0.007	0.008	0.799
Liver, polar lipids	C20:5(n-3)	0.023	0.008	0.004	0.616
	n6/n3	− 0.279	0.104	0.007	0.997

**Table 6:** Association results for polymorphism in gene *NLE1* (168_NLE1, position 40,037,354). Traits with *p-*value < 0.01 are shown and those with q < 0.10 are highlighted in bold.

Timepoint/Location	Trait	b	SE	*p-*value	*q-*value
Slaughter	Liver fat content	1.018	0.313	0.001	0.215
Backfat, inner layer	C16:0	0.426	0.140	0.002	0.852
	**C18:0**	**1.508**	**0.271**	**2.64E−08**	**2.64E−05**
	C18:1(n-9)	− 0.617	0.185	8.63E−04	0.404
	SFA	0.898	0.257	4.68E−04	0.235
	MUFA	− 0.787	0.222	3.84E−04	0.193
	UI	− 0.008	0.003	0.005	0.853
	**MUFA/SFA**	**− 0.058**	**0.015**	**1.57E−04**	**0.079**
Backfat, outer layer	C16:0	0.344	0.121	0.004	0.557
	MUFA	− 0.595	0.193	0.002	0.413
	MUFA/SFA	− 0.036	0.013	0.006	0.526
*Longissimus dorsi* muscle, neutral lipids	**C16:0**	**1.016**	**0.251**	**5.08E−05**	**0.025**
	C16:1(n-7)	− 0.251	0.081	0.002	0.168
	C18:0	0.444	0.128	0.001	0.350
	**C18:1(n-9)**	**− 0.930**	**0.246**	**1.57E−04**	**0.079**
	C18:1(n-7)	− 0.209	0.068	2.11E−03	0.350
	**SFA**	**1.481**	**0.368**	**5.82E−05**	**0.029**
	**MUFA**	**− 1.400**	**0.346**	**5.20E−05**	**0.026**
	**UI**	**− 1.584**	**0.410**	**1.14E−04**	**0.057**
	**C18:1/C18:0**	**− 0.353**	**0.091**	**9.98E−05**	**0.072**
	**MUFA/SFA**	**− 0.116**	**0.028**	**4.27E−05**	**0.021**

Three different SNPs located in *SCD* gene were associated with palmitoleic acid and the desaturation ratio oleic/stearic only in the neutral lipid fraction of liver (C16:1n-9, C18:1/C18:0) (Tables 2, 4). However, significant effects at the nominal level were observed for inner backfat FA traits, which did not reach the multiple-test corrected significance threshold (Supplementary Table S6). Also, the key genes of lipogenesis, *FASN* and *ACACA,* were associated with miristic acid (C14:0) and palmitoleic acid (C16:1n-7), respectively, in both the backfat outer layer and the neutral lipids from muscle (Table 4).

Besides, some other relevant genes known to influence fatness related traits, such as *IRX3*, *ADIPOQ*, *MSTN* or *MYOD1*, had significant effects on just one trait (Supplementary Table S5).

#### Blood biochemical parameters

In relation to metabolic traits, only two significant associations were found for HDL concentration in plasma at 110 and 215 days-old, both related to WGS markers (Supplementary Table S5). The association found at 110 days old involves a SNP located in *TBC1D16* gene, while the association found at 215 days-old involves the gene *CD4*.

No significant effect was observed for any of the other meat quality traits included in the study (intramuscular fat content, pH and drip loss) after the multiple test correction.

## Discussion

In this study, a medium-throughput genotyping approach was performed after an initial SNP discovery step, in order to uncover DNA polymorphisms related to relevant productive traits. SNPs analysed were mostly obtained from WGS data, and complemented with a panel of biological and functional candidate genes/SNPs, obtained from literature and RNAseq data.

Genotyping results showed a good level of informativity of the markers derived from WGS, with a high proportion of markers showing intermediate frequencies (56% showed 0.3 < MAF < 0.7) and scarce proportion being discarded due to low segregation (10.6%). These results support the validity of the SNP discovery approach perfomed from WGS data. On the contrary, markers obtained from bibliography and RNAseq data were less informative (33% showed 0.3 < MAF < 0.7) and a high proportion was detected as monomorphic or scarcely informative (50%). This finding was not unexpected as WGS markers were obtained from sequencing data from animals of the same Iberian crossbred population employed for the association study, while candidate genes/SNPs were previously identified in different pig breeds and populations.

In spite of a lower number of previously known candidate SNPs, and their lower informativity, in comparison to WGS-derived markers (102 vs 903) a much higher number of significant associations were observed for the SNPs with previous evidences of association or differential expression. This is not surprising as those fulfill more functional criteria than the ones discovered in the present work from WGS data. In fact, out of 174 significant associations, 152 involved previously identified SNPs in candidate genes and only 22 involved WGS-derived markers. Besides, out of the 152 associations related to previously characterized SNPs, 62 corresponded to well-known markers with previous evidences of association, and 90 corresponded to markers discovered from RNAseq data, within genes showing differential expression between Iberian pig genotypes or with a potential regulatory role. This finding supports the usefulness of deep sequencing transcriptome data mining to uncover structural variants with interesting effects on phenotype.

Regarding growth and development traits, two main genes, *ACACA* and *SCD*, were found to affect, respectively, birth measures and livelong performance traits. These genes are involved in fat synthesis and metabolism and, indeed, significant effects were also found for both genes on FA composition. However, their potential role on growth traits is not clear so far. *Acetyl-CoA carboxylase* (*ACACA*) is a multifunctional enzyme system that catalyzes the carboxylation of acetyl-CoA to malonyl-CoA, the rate-limiting step in FA synthesis. In agreement, our results show a significant effect of this gene on palmitoleic acid abundance in backfat and muscle and suggestive effects on many main FA and FA ratios. Besides this main role, the *ACACA* gene influences mitochondrial membrane potential, thus it might condition cell proliferation and tissue growth^44^. In fact, a deficiency in *ACACA* activity was associated to defects in early growth and muscle development in humans^45^. *Stearoyl-CoA desaturase* (*SCD*) catalyzes the ∆9-cis desaturation of palmitoyl- and stearoyl-CoA, which are converted into palmitoleoyl- and oleoyl-CoA, respectively. In addition to being components of tissue lipids, MUFAs also serve as mediators of signal transduction, cellular differentiation, food intake, apoptosis, and mutagenesis, and therefore, variation in *SCD* function in mammals would be expected to have an effect on a variety of key physiological events, including growth^46^. Thus, it has been proposed that BW gain may be associated with *SCD* increased activity. In fact, a different polymorphism in *SCD* gene was associated with ADG and FCR in lean pigs^47^. Nevertheless, the three SNPs analysed in this study (g.2108C>T; g.2228T>C; g.2281A>G), located in the promoter region and in full linkage disequilibrium, had not been previously associated with performance traits^48–50^. This novel finding is also striking because of the size of the effect, especially on BW traits, with an estimated additive effect close to 30 kg. This finding should be cautiously interpreted, as the frequency of the homozygous genotype was low and the effect could be overestimated. Interestingly, Estany et al*.*^48^ found that C-T-A haplotype was additively associated with enhanced C18∶1/C18∶0 desaturation index both in muscle and subcutaneous fat, but not in liver, in a purebred Duroc line. According to our results, the main effect of *SCD* gene regarding FA composition is detected in liver neutral lipid fraction, with C-T-A haplotype enhancing the desaturation index as well as C16:1n-9 content. Also, a potential effect on IMF was reported for this mutation^49^ which has not been validated in the present work.

Other interesting genes and SNPs that have not yet been established as candidates for growth traits were also identified. Some of them were obtained from RNAseq data and were differentially expressed between Iberian and Iberian × Duroc crossbred or Duroc purebred pigs, such as *FREM2*, *EPHX1*, *SOWAHB* or *TFRC*^5,13–15^. Thus, those genes fulfill a functional criteria to be considered involved in the phenotypic differences observed between Iberian and Duroc genotypes. The *FREM2* gene (*FRAS1 related extracellular matrix protein 2*) encodes an extracellular matrix protein with a role in morphogenetic processes^51^. In agreement with our findings, the *FRAS/FREM* complex is particularly important during early development. In humans, mutations in this gene cause the Fraser syndrome, a multisystem disorder involved in the structural adhesion of the skin ephitelium to its underlying mesenchyma^52^ and in pigs this gene has been proposed as candidate for developmental morphological traits such as presence of wattles^53^, The genes *SOWAHB* and *EPHX1* showed somewhat similar association results, both affecting livelong growth traits and some fat deposition traits. The gene *SOWAHB* (*sosondowah ankyrin repeat domain family member B*) is part of the Ankyrin family, involved in membrane skeleton organisation, ionic transport, protein recognition as well as cell–cell adhesion regulation^54^. Moreover, *SOWAHB* is activated in response to insulin and possibly involved in insulin resistance^55^. Besides its effect on growth traits, significant nominal *p*-values were observed in the test of association with fatness traits at different ages (backfat thickness, cholesterol, Supplementary Table S6) suggesting a role in lipid metabolism. This gene showed 4 × higher expression in loin of purebred Iberian than in crossbred growing pigs^14^. The *EPHX1* (*microsomal epoxide hydrolase*) gene showed significant association results for growth as well as FA composition traits, specifically for C20:1(n-9) and for C20:3(n-6) contents. The enzyme encoded by this gene is able to either detoxify or bioactivate a wide range of substrates^56^ including epoxides derived from endogenous polyunsaturated FA, thus mediating several biological processes such as inflammation or angiogenesis, and regulating crucial signaling pathways for cellular homeostasis, adipocyte differentiation and insulin response^57–59^. This gene was shown to be upregulated in *biceps femoris*, *longissimus dorsi* and backfat of pure Iberians in comparison to Duroc genotypes^13–15^. The upregulation of *SOWAHB* and *EPHX1* genes in pure Iberians in different tissues may be related to their potential role in insulin response and adiposity, which is in agreement with the suggestive association of their structural variants with fatness traits and significant association with weight at growing stages, when Iberian pigs are known to develop a compensatory growth and a more intense development than Duroc genotypes^5^. The *TFRC* gene (*Transferrin Receptor*) encodes a cell surface receptor necessary for cellular iron uptake and has been studied as a positional candidate gene for disease susceptibility^60^. In addition, it acts as a lipid sensor that regulates mitochondrial function by regulating activation of the JNK pathway^61^. Interestingly, *TFRC* was upregulated in loin muscle of Duroc × Iberian crossbred pigs at birth^14^, but it was upregulated in *biceps femoris* and backfat of Iberian purebred growing pigs^5,15^. According to our results, the SNP in this gene affects BW and ADG. Thus, the opposite regulation observed at birth and growing stages would be related to the different paucity of muscle developmental processes observed in Iberian vs Duroc genotypes, with the latter showing higher prenatal development and birth weight and the Iberian purebreds showing increased muscle development at growing stages.

Newborn developmental traits were also affected by several SNPs detected through WGS, located in relatively unknown genes such as *LRIG3*, *DENND1B* or *TMCC1*. Although none of these genes has been studied for association with any productive trait, all have roles or evidences which suggest involvement in early developmental processes. *LRIG3* (*leucine-rich repeats and immunoglobulin-like domains 3*) plays a role in embryo development, including cranio-facial morphogenesis and neural crest formation^62^. Also a direct relationship for *LRIG3* and early growth was observed since KO mice were smaller than wild-type ones^63^. In agreement, our results show significant effects on birth weight and thoracic circumference and suggestive effects on all remaining traits recorded in neonates, including those related to head development. *DENN Domain Containing 1B* (*DENND1B*) gene has an important role on cytokine production and regulation of T cell receptor signaling and mutations or loss of this factor were associated with immune diseases in neonates^64^. Interestingly, methylation of *DENND1B* gene in venous umbilical cord blood at delivery has been associated to birth weight in humans^65^. According to our results, this gene influences birth weight, abdominal circumference and biparietal diameter and is suggestively associated to most early traits recorded at birth and at weaning. *TMCC1* (*Transmembrane And Coiled-Coil Domain Family 1)* is an integral endoplasmic reticulum (ER) membrane protein that has been associated with central adiposity and waist circumference^66^ and is supposed to be involved in regulation of myogenesis^67^. Our results indicate effects on biparietal and thoracic diameter as well as thoracic circumference, and also at a suggestive level, on several other birth traits and later fatness traits such as backfat.

Fattening traits were mainly affected by SNPs located in known candidate genes such as *LEPR*, *LIPE* or *FTO*, with the clearest results detected for *LEPR*. Leptin receptor is known to have a determinant role on energy homeostasis and has been widely studied as functional and positional candidate gene for fat deposition traits. Iberian pigs are known to carry a fixed mutation in this gene which is considered a causal mutation contributing to their characteristic trend for adiposity and high appetite as well as their leptin resistant phenotype^7,8^. This mutation has been evaluated in different genetic backgrounds, mainly in experimental pig populations, but also in other breeds and commercial populations^50,68–70^. According to the present results, the effects of this SNP on fatness are further validated in our Iberian × Duroc commercial population, with significant effects on different backfat thickness measures and global ADG, and suggestive associations with BW, FA composition and cholesterol, HDL and LDL levels. As expected, the Iberian T allele increases fattening, with an additive effect close to 0.5 cm for backfat, which means an estimated difference of almost 1 cm between homozygous animals for alternative alleles (20% of the trait mean value).

Besides the known candidate genes, the *NFE2L2* gene (*nuclear factor erythroid 2-related factor 2*) was associated with fatness. This gene codes a transcription factor whose main role involves the regulation of the expression of antioxidant proteins that protect against oxidative damage. Moreover, this transcription factor is determinant in the maintenance of homeostasis, participating in the regulation of metabolism, inflammation, autophagy, proteostasis, mitochondrial physiology, and immunity^71^. This gene was selected from RNAseq data for SNP discovery, despite not being differentially expressed, due to its potential role as predicted regulator for the differential expression observed between Iberian pig genotypes^14^. Our results show a significant effect on backfat thickness and suggestive effects on intramuscular fat content and FA composition, especially in loin, in agreement with its known role in muscle mitochondrial biogenesis and the predicted role as regulator of muscle metabolism.

The main genes affecting FA composition were *KRT10* and *NLE1*, both affecting the proportions of SFA and MUFA in backfat and loin. *KRT10* (*keratin 10*) was studied because it was overexpressed in muscle from pure Iberian in comparison to crossbred pigs^13^, although it does not have a known role in relation to fat metabolism. However, it has been recently shown that *KRT10* expression is suppressed by serum lipids^72^ and, interestingly, different keratin genes had been associated to FA composition traits in a chromosome 12 survey in Iberian × Landrace backcrossed pigs^73^. In fact, the *KRT10* gene maps on SSC12, where several QTL for FA composition traits have been discovered. Moreover, *KRT10* was found as the most significant differentially expressed gene according to *ACACA* genotype regarding polymorphism ALGA0066302A, not analysed here, which in turn is associated with FA composition. On the other hand, the *NLE1* gene (*Notchless homolog 1*) plays a role in regulating the Wnt pathway, which is known to play a central role in adipocyte differentiation and lipid metabolism^74^. In fact, Wnt pathway plays a dual function in adipocytes, including a well-known repressive effect on adipogenesis and the stimulation of leptin production in mature adipocytes^75^. In agreement with the antiadipogenic effect, a higher expression of this gene was observed in loin of crossbred Iberian pigs, which are leaner than purebreds^14^. Our association results do not relate this gene with adiposity traits, but clearly demonstrates its involvement in relevant traits related to FA metabolism and profile, which influence meat quality, in the two main productive tissues, fat and muscle, and especially on this last one. The allele that increases oleic acid content and monounsaturated to saturated fat proportions, with effects ranging from 0.5 to 1 SD in magnitude, also shows suggestive positive effects on BW and ADG at late growing stages, meaning it could be an interesting marker to improve meat quality without negative pleiotropic effects on other relevant traits.

Several other SNPs showed significant effects limited to a few FA. *KCNH2* gene (*Potassium Voltage-Gated Channel Subfamily H Member 2*) was associated with the main PUFA contents in backfat, although, suggestive effects were observed on the muscle FA profile and on some growth and carcass traits. This gene was previously found overexpressed in loin and *biceps* of pure Iberians in comparison to crossbreds and Duroc animals^5,14^. Our results agree with previous findings in humans showing that mutations in *KCNH2* gene are associated with alteration of insulin homeostasis and glucose and lipid metabolism^76^ and its methylation is related to obesity^77^. *AHNAK* gene (*Neuroblast differentiation-associated protein*) codes a nucleoprotein involved in adipogenesis and lipid metabolism. *AHNAK* KO mice have reduced fat accumulation and decreased serum triglyceride levels as well as increased expression of genes involved in lipolysis and FA oxidation^78^. In agreement, its expression was higher in pure Iberian animals^14^, which are characterised by a higher adipogenic potential than crossbreds. In addition, our results show effects of this SNP on different FA in backfat and liver and a suggestive effect on backfat thickness at weaning.

On the other hand, the genes *FASN*, *LIPE* and *TNF* had similar effects, mainly on C14:0 in backfat and muscle. As mentioned previously, *LIPE* gene (*hormone sensitive lipase*) was one of the genes significantly affecting backfat depth at slaughter. The *LIPE* allele with a positive effect on backfat also positively affected C14:0 and C16:0 in backfat and loin and had suggestive negative effects on several MUFA parameters and glucose metabolism indicators. *LIPE* is a key enzyme in the mobilization of FA from acylglycerols^79^, which has been related to different fat deposition and FA profile traits in different species. On the other hand, *FASN* gene (*fatty acid synthase*) codes a multifunctional enzyme that catalyzes the synthesis of FA and has been repeatedly associated with FA composition traits, especially affecting short chain saturated FA^21,80^, as in our results. This gene, together with *ACACA*, regulates de novo synthesis of FA. Thus, *FASN*, *ACACA* and *LIPE* are involved in the regulation of fat deposition by balancing lipogenesis and lipolysis, and significant association results are obtained for all three of them. On the other hand, tumor necrosis factor (*TNF*) is an adipokine that promotes insulin resistance and is associated with obesity-induced type 2 diabetes^81^. In agreement, our results indicate a significant effect on C14:0 in backfat and loin, but also many other suggestive associations are found for other saturated and monounsaturated FA, as well as carcass and meat quality traits (backfat thickness at slaughter, drip loss) and BW records along growth.

Scarce significant associations were found for metabolic traits. Only HDL concentration was significantly associated with two different SNPs, both derived from WGS data, at two different ages. The association found at 110 days old is interesting as it involves *TBC1D16* gene, which has a role in membrane trafficking and molecule transport and is known to be dysregulated in obesity^82^. Besides the significant effect on HDL, this SNP showed suggestive effects on many different FA traits related to MUFA contents and MUFA/SFA ratios in loin and liver, which are known to be correlated with physical markers of obesity such as body mass and adiposity indexes^83^, in agreement with the role of this gene, although no effect was observed on fatness.

## Conclusions

A combined approach of candidate SNP recovery from literature and SNP discovery from WGS and RNAseq data has been successful in validating the effects of different SNPs in candidate genes previously associated with fatness, growth and FA composition traits (such as *LEPR*, SCD, *ACACA, FASN or LIPE*). Moreover, our approach allowed for the discovery of interesting new effects of SNPs in less known genes (such as *LRIG3*, *DENND1B*, *NMNAT1*, *SOWAHB, EPHX1*) affecting BW and ADG at different ages, as well as fatness and FA composition (*NFE2L2, KRT10* or *NLE1*). Most significant associations came from SNPs identified through structural mining of transcriptome deep-sequencing data. In spite of a relatively small number of significant associations detected for WGS-derived markers, the approach employed here is proposed as a useful genome-wide strategy for the discovery of untapped genetic basis of productive traits. Our results contribute to a better understanding of the molecular genetic basis of relevant traits in pigs, including both growth and yield-related traits as well as traits involved in sensory and technological quality of meat which are essential for Iberian pig cured products. Besides, our results provide interesting SNPs and new candidate genes for association studies in different pig breeds and populations and for future implementation of marker-assisted selection strategies. Different markers can be highlighted as most promising, such as *KRT10*, *NLE1*, *TFRC*, *NFE2L2*, *SCD* or *LEPR*, and some of them may allow the improvement of meat quality without negatively affecting growth efficiency, or viceversa.

## Supplementary Information

Supplementary Legends.

Supplementary Table S1.

Supplementary Table S2.

Supplementary Table S3.

Supplementary Table S4.

Supplementary Table S5.

Supplementary Table S6.

## Data Availability

The authors confirm that the data supporting the findings of this study are available within the article and its supplementary materials. The raw WGS datasets generated during the current study have been deposited in the DDBJ Sequence Read Archive (DRA) repository under accession number DRA014083.

## References

[1] Lopez-Bote CJ (1998). Sustained utilization of the Iberian pig breed. Meat Sci..

[2] Rey AI, Daza A, López Carrasco C, López-Bote CJ (2006). Feeding Iberian pigs with acorn and grass in either free-range or confinement affects the carcass characteristics and fatty acids and tocopherols accumulation in *Longissimus dorsi* muscle and backfat. Meat Sci..

[3] Juárez M, Clemente I, Polvillo O, Molina A (2009). Meat quality of tenderloin from Iberian pigs as affected by breed strain and crossbreeding. Meat Sci..

[4] Clemente I, de Pedro EJ, Cabezas AB (2012). Comparison of pork quality from pure and crossbred Iberian pig. 7th International Symposium on the Mediterranean Pig. Options Méditerranéennes: Série A Séminaires Méditerranées, n. 101.

[5] Benítez R (2021). Changes in *biceps femoris* transcriptome along growth in Iberian pigs fed different energy sources and comparative analysis with Duroc breed. Animals.

[6] Vázquez-Gómez M (2020). Piglet birth weight and sex affect growth performance and fatty acid composition in fatty pigs. Anim. Prod. Sci..

[7] Ovilo C (2005). Fine mapping of porcine chromosome 6 QTL and LEPR effects on body composition in multiple generations of an Iberian by Landrace intercross. Genet Res..

[8] Óvilo C (2010). Hypothalamic expression of porcine leptin receptor (LEPR), neuropeptide Y (NPY), and cocaine- and amphetamine-regulated transcript (CART) genes is influenced by LEPR genotype. Mamm. Genome..

[9] Corominas J (2013). Polymorphism in the ELOVL6 gene is associated with a major QTL effect on fatty acid composition in pigs. PLoS One.

[10] Corominas J (2012). Evaluation of the porcine ACSL4 gene as a candidate gene for meat quality traits in pigs. Anim. Genet..

[11] Mercadé A (2006). Characterization of the porcine acyl-CoA synthetase long-chain 4 gene and its association with growth and meat quality traits. Anim. Genet..

[12] Fernández-Barroso MÁ (2020). Genetic parameter estimation and gene association analyses for meat quality traits in open-air free-range Iberian pigs. J. Anim. Breed. Genet..

[13] Ayuso M (2015). Comparative analysis of muscle transcriptome between pig genotypes identifies genes and regulatory mechanisms associated to growth, fatness and metabolism. PLoS One.

[14] Ayuso M (2016). Developmental stage, muscle and genetic type modify muscle transcriptome in pigs: Effects on gene expression and regulatory factors involved in growth and metabolism. PLoS One.

[15] Benítez R (2019). Breed, diet, and interaction effects on adipose tissue transcriptome in Iberian and Duroc pigs fed different energy sources. Genes.

[16] Van Son M (2017). Genome-wide association study confirm major QTL for backfat fatty acid composition on SSC14 in Duroc pigs. BMC Genom..

[17] Zhang W (2016). Genome-wide association studies for fatty acid metabolic traits in five divergent pig populations. Sci. Rep..

[18] Ramayo-Caldas Y (2012). Genome-wide association study for intramuscular fatty acid composition in an Iberian × Landrace cross. J. Anim. Sci..

[19] Muñoz M (2013). Genome-wide analysis of porcine backfat and intramuscular fat fatty acid composition using high-density genotyping and expression data. BMC Genom..

[20] Martínez-Montes ÁM (2018). Using genome wide association studies to identify common QTL regions in three different genetic backgrounds based on Iberian pig breed. PLoS One.

[21] Crespo-Piazuelo D (2020). Identification of strong candidate genes for backfat and intramuscular fatty acid composition in three crosses based on the Iberian pig. Sci. Rep..

[22] Pena RN (2019). Five genomic regions have a major impact on fat composition in Iberian pigs. Sci. Rep..

[23] Muñoz M (2019). Genomic diversity, linkage disequilibrium and selection signatures in European local pig breeds assessed with a high density SNP chip. Sci. Rep..

[24] Daetwyler HD (2014). Whole-genome sequencing of 234 bulls facilitates mapping of monogenic and complex traits in cattle. Nat. Genet..

[25] de Blas, C., Gasa, J. & Mateos, G. G. Necesidades nutricionales para ganado porcino. Normas FEDNA. Fundación Española para el Desarrollo de la Nutrición Animal. http://fundacionfedna.org/sites/default/files/Normas%20PORCINO_2013rev2_0.pdf (2013).

[26] Calvo L, Toldrá F, Aristoy MC, Lopez-Bote CJ, Rey AI (2016). Effect of dietary organic selenium on muscle proteolytic activity and water-holding capacity in pork. Meat Sci..

[27] Segura J, Lopez-Bote CJ (2014). A laboratory efficient method for intramuscular fat analysis. Food Chem..

[28] Hulbert AJ, Pamplona R, Buffenstein R, Buttemer WA (2007). Life and death: Metabolic rate, membrane composition, and life span of animals. Physiol. Rev..

[29] Hulver MW (2005). Elevated stearoyl-CoA desaturase-1 expression in skeletal muscle contributes to abnormal fatty acid partitioning in obese humans. Cell Metab..

[30] Bolger AM, Lohse M, Usadel B (2014). Trimmomatic: A flexible trimmer for Illumina Sequence Data. Bioinformatics.

[31] Warr A (2020). An improved pig reference genome sequence to enable pig genetics and genomics research. GigaScience.

[32] Li H, Durbin R (2009). Fast and accurate short read alignment with Burrows–Wheeler transform. Bioinformatics.

[33] Li H (2009). The sequence alignment/map format and SAMtools. Bioinformatics.

[34] Poplin R (2018). Scaling accurate genetic variant discovery to tens of thousands of samples. bioRxiv..

[35] Van der Auwera GA, O'Connor BD (2020). Genomics in the Cloud: Using Docker, GATK, and WDL in Terra.

[36] Danecek P (2011). 1000 Genomes project analysis group. The variant call format and VCFtools. Bioinformatics.

[37] McLaren W (2016). The Ensembl variant effect predictor. Genome Biol..

[38] Kumar P (2009). Predicting the effects of coding non-synonymous variants on protein function using the SIFT algorithm. Nat. Protoc..

[39] Chang C (2015). Second-generation PLINK: Rising to the challenge of larger and richer datasets. GigaScience.

[40] Ayuso, M. *et al.* Identification and prioritization of SNPs potentially involved in transcriptomic and phenotypic differences between pure and crossbred Iberian pigs. 36th International Society for Animal Genetics (ISAG) Conference, Abstract Book, p. 128 (2017).

[41] Yang (2011). GCTA: A tool for genome-wide complex trait analysis. Am. J. Hum. Genet..

[42] Storey, J. D., Bass, A. J., Dabney, A. & Robinson, D. Qvalue: Q-value estimation for false discovery rate control. R package version 2.16.0. http://github.com/jdstorey/qvalue (2019).

[43] RStudio Team. RStudio: Integrated development for R. http://www.rstudio.com/ (RStudio, PBC, 2020).

[44] Zhang H (2021). Down-regulation of *ACACA* suppresses the malignant progression of prostate cancer through inhibiting mitochondrial potential. J. Cancer.

[45] Blom W, de MuinckKeizer SMPF, Stolte HR (1981). Acetyl-CoA carboxylase deficiency: An inborn error of de novo fatty acid synthesis. N. Engl. J. Med..

[46] Ntambi JM, Ntambi J (2013). Stearoyl-CoA desaturase-1 is a biological regulator of energy homeostasis. Stearoyl-CoA Desaturase Genes in Lipid Metabolism.

[47] Bartz M, Kociucka B, Salamon S, Jelen H, Switonski M (2012). Transcript level of the pig SCD gene has no effect on fatty acid composition in muscle and fat tissues, but its polymorphism within the putative miRNA target site is associated with daily gain and feed conversion ratio. J. Anim. Sci..

[48] Estany J, Ros-Freixedes R, Tor M, Pena RN (2014). A functional variant in the stearoyl-CoA desaturase gene promoter enhances fatty acid desaturation in pork. PLoS One.

[49] Fernández AI (2017). Validating porcine SCD haplotype effects on fatty acid desaturation and fat deposition in different genetic backgrounds. Livest. Sci..

[50] Ros-Freixedes R (2016). Genome-wide association study singles out SCD and LEPR as the two main loci influencing intramuscular fat content and fatty acid composition in Duroc pigs. PLoS One.

[51] Timmer JR (2005). Tissue morphogenesis and vascular stability require the Frem2 protein, product of the mouse myelencephalic blebs gene. Proc. Natl. Acad. Sci. U.S.A..

[52] Petrou PP, Makrygiannis AK, Chalepakis G (2008). The Fras1/Frem family of extracellular matrix proteins: Structure, function, and association with fraser syndrome and the mouse bleb phenotype. Connect. Tissue Res..

[53] Schiavo G (2019). Genome-wide association analyses for several exterior traits in the autochthonous Casertana pig breed. Livest. Sci..

[54] Hryniewicz-Jankowska A, Czogalla A, Bok E, Sikorsk AF (2002). Ankyrins, multifunctional proteins involved in many cellular pathways. Folia Histochem. Cytobiol..

[55] Haider N, Mann M, Kahn CR (2021). Signaling defects associated with insulin resistance in nondiabetic and diabetic individuals and modification by sex. J. Clin. Investig..

[56] Václavíková R, Hughes DJ, Souček P (2015). Microsomal epoxide hydrolase 1 (EPHX1): Gene, structure, function, and role in human disease. Gene.

[57] McReynolds C, Morisseau C, Wagner K, Hammock B (2020). Epoxy fatty acids are promising targets for treatment of pain, cardiovascular disease and other indications characterized by mitochondrial dysfunction, endoplasmic stress and inflammation. Adv. Exp. Med. Biol..

[58] Gautheron J (2021). EPHX1 mutations cause a lipoatrophic diabetes syndrome due to impaired epoxide hydrolysis and increased cellular senescence. Elife.

[59] Edin ML (2018). Epoxide hydrolase 1 (EPHX1) hydrolyzes epoxyeicosanoids and impairs cardiac recovery after ischemia. J. Biol. Chem..

[60] Jacobsen M (2011). Characterisation of five candidate genes within the ETEC F4ab/ac candidate region in pigs. BMC Res. Notes.

[61] Senyilmaz D (2015). Regulation of mitochondrial morphology and function by stearoylation of TFR1. Nature.

[62] Abraira VE (2008). Cross-repressive interactions between *LRIG3* and netrin 1 shape the architecture of the inner ear. Development.

[63] Hellström M (2016). Cardiac hypertrophy and decreased high-density lipoprotein cholesterol in Lrig3-deficient mice. Am. J. Physiol. Regul. Integr. Comp. Physiol..

[64] Yang CW (2016). Regulation of T cell receptor signaling by DENND1B in TH2 cells and allergic disease. Cell.

[65] Agha G (2016). Birth weight-for-gestational age is associated with DNA methylation at birth and in childhood. Clin. Epigenet..

[66] Yoneyama S (2014). Gene-centric meta-analyses for central adiposity traits in up to 57 412 individuals of European descent confirm known loci and reveal several novel associations. Hum. Mol. Genet..

[67] Harada A (2015). Spatial re-organization of myogenic regulatory sequences temporally controls gene expression. Nucleic Acids Res..

[68] Pérez-Montarelo D (2012). Joint effects of porcine leptin and leptin receptor polymorphisms on productivity and quality traits. Anim. Genet..

[69] Galve A (2012). The effects of leptin receptor (LEPR) and melanocortin-4 receptor (MC4R) polymorphisms on fat content, fat distribution and fat composition in a Duroc × Landrace/Large White cross. Livest. Sci..

[70] Uemoto Y (2012). Effects of porcine leptin receptor gene polymorphisms on backfat thickness, fat area ratios by image analysis, and serum leptin concentrations in a Duroc purebred population. Anim. Sci. J..

[71] He F, Ru X, Wen T (2020). NRF2, a transcription factor for stress response and beyond. Int. J. Mol. Sci..

[72] Aldehlawi H (2020). Serum lipids, retinoic acid and phenol red differentially regulate expression of keratins K1, K10 and K2 in cultured keratinocytes. Sci. Rep..

[73] Muñoz M (2012). Survey of SSC12 regions affecting fatty acid composition of intramuscular fat using high-density SNP data. Front. Genet..

[74] Caputo T (2021). Anti-adipogenic signals at the onset of obesity-related inflammation in white adipose tissue. Cell. Mol. Life Sci..

[75] Chen ZL (2015). Acute Wnt pathway activation positively regulates leptin gene expression in mature adipocytes. Cell Signal..

[76] Engelbrechtsen L (2018). Common variants in the hERG (KCNH2) voltage-gated potassium channel are associated with altered fasting and glucose-stimulated plasma incretin and glucagon responses. BMC Genet..

[77] Sonne SB (2017). Obesity is associated with depot-specific alterations in adipocyte DNA methylation and gene expression. Adipocyte.

[78] Kim YN (2021). Ahnak deficiency attenuates high-fat diet-induced fatty liver in mice through FGF21 induction. Exp. Mol. Med..

[79] Lampidonis AD, Rogdakis E, Voutsinas GE, Stravopodis DJ (2011). The resurgence of HormoneSensitive Lipase (HSL) in mammalian lipolysis. Gene.

[80] Zappaterra M (2019). Association study between backfat fatty acid composition and SNPs in candidate genes highlights the effect of FASN polymorphism in large white pigs. Meat Sci..

[81] Sethi JK, Hotamisligil GS (2021). Metabolic messengers: Tumour necrosis factor. Nat. Metab..

[82] Jacobsen MJ (2019). Epigenetic and transcriptomic characterization of pure adipocyte fractions from obese pigs identifies candidate pathways controlling metabolism. Front. Genet..

[83] Jeyakumar SM (2009). Fatty acid desaturation index correlates with body mass and adiposity indices of obesity in Wistar NIN obese mutant rat strains WNIN/Ob and WNIN/GR-Ob. Nutr. Metab. (Lond.).

