# Supplementary material for: SNP discovery and association study for growth, fatness and meat quality traits in Iberian crossbred pigs
Source: Sci Rep. 2022 Sep 30;12:16361. doi: 10.1038/s41598-022-20817-0 (PMC9525691; doi:10.1038/s41598-022-20817-0)
Supplement: Supplementary file 1 — Supplementary Legends. [file 41598_2022_20817_MOESM1_ESM.docx]

**Supplementary material**

Supplementary table S1. List and details of analysed markers selected from WGS data and genotyped by genotyping by sequencing (GBS).

Supplementary table S2. List and details of analysed markers selected from literature and RNAseq data and genotyped by openarray (OA).

Supplementary table S3. Fixed effects and covariates included in the model for the association analyses of the different groups of traits.

Supplementary table S4. Descriptive statistics for the analysed phenotypic traits.

Supplementary table S5. Significant association results (q<0.10)

Supplementary table S6. Association results with significant nominal p-values (p<0.05), for those genes showing at least one significant association (q<0.10).
